# Supplementary material for: Determinants of Alcohol Consumption among Medical Students: Results from POLLEK Cohort Study
Source: Int J Environ Res Public Health. 2021 May 30;18(11):5872. doi: 10.3390/ijerph18115872 (PMC8199068; doi:10.3390/ijerph18115872)
Supplement: Supplementary file 1 [file ijerph-18-05872-s001.zip › ijerph-1227040-supplementary.pdf]

International Journal of
*Environmental Research and Public Health*

MDPI

**Table S1.** Full version of the analysis of alcohol use regarding selected personal characteristics. Part A: Qualitative variables with numbers and frequencies (in brackets) and *p*-values. Part B: Quantitative variables with medians (Me) and interquartile ranges (IQR) in brackets and *p*-values. Missing values were not shown.

**Part A: Qualitative Variables**

**Number (frequency) with the *p*-value (*p*)**

| Variable | Value | AUDIT (*n* = 540) | | | AUDIT C (N = 544) | | |
|---|---|---|---|---|---|---|---|
| | | HAZ *n* = 167 (30.9%) | Low risk *n* = 373 (69.1%) | *p* | HAZ *n* = 188 (34.6%) | Low risk *n* = 356 (65.4%) | *p* |
| Gender | Female | 71 (21.4%) | 261 (78.6%) | <0.001 | 78 (23.4%) | 255 (76.6%) | <0.001 |
| | Male | 96 (46.2%) | 112 (53.8%) | | 110 (52.1%) | 101 (47.9%) | |
| Faculty | Katowice | 115 (34.2%) | 221 (65.8%) | 0.03 | 133 (39.2%) | 206 (60.8%) | 0.004 |
| | Zabrze | 52 (25.5%) | 152 (74.5%) | | 55 (26.8%) | 150 (73.2%) | |
| Source of income-dependent on parents | Yes | 165 (31.9%) | 352 (68.1%) | 0.02 | 186 (35.8%) | 334 (64.2%) | 0.004 |
| | No | 2 (8.7%) | 21 (91.3%) | | 2 (8.3%) | 22 (91.7%) | |
| Smoking cigarette (3 categories) | Never | 62 (19.7%) | 253 (80.3%) | <0.001 | 76 (23.9%) | 242 (76.1%) | <0.001 |
| | Former | 67 (45.3%) | 81 (54.7%) | | 74 (49.7%) | 75 (50.3%) | |
| | Current | 38 (49.4%) | 39 (50.6%) | | 38 (49.4%) | 39 (50.6%) | |
| Smoking cigarette (2 categories) | Never | 62 (19.7%) | 253 (80.3%) | <0.001 | 76 (23.9%) | 242 (76.1%) | <0.001 |
| | Ever | 105 (46.7%) | 120 (53.3%) | | 112 (49.6%) | 114 (50.4%) | |
| E-cigarettes (3 categories) | Never | 73 (20.4%) | 285 (79.6%) | <0.001 | 88 (24.4%) | 273 (75.6%) | <0.001 |
| | Former | 61 (45.5%) | 73 (54.5%) | | 67 (50.0%) | 67 (50.0%) | |
| | Current | 29 (69.0%) | 13 (31.0%) | | 28 (65.1%) | 15 (34.9%) | |
| E-cigarettes (2 categories) | Never | 73 (20.4%) | 285 (79.6%) | <0.001 | 88 (24.4%) | 273 (75.6%) | <0.001 |
| | Ever | 90 (51.1%) | 86 (48.9%) | | 95 (53.7%) | 82 (46.3%) | |
| Under surveillance due to diagnosis of chronic disease | Yes | 19 (26.4%) | 53 (73.6%) | 0.03 | 23 (31.5%) | 50 (68.5%) | NS |
| | No | 17 (50.0%) | 17 (50.0%) | | 14 (41.2%) | 20 (58.8%) | |
| Take up sports activities to improve physical fitness | At least 3 times a week for 30 min | 43 (37.1%) | 73 (62.9%) | 0.1 | 49 (41.5%) | 69 (58.5%) | 0.1 |
| | Less often | 100 (30.6%) | 227 (69.4%) | | 111 (33.7%) | 218 (66.3%) | |
| | Not at all | 24 (24.7%) | 73 (75.3%) | | 28 (28.9%) | 69 (71.1%) | |
| Take up sports activities to improve physical fitness (2 categories) | Yes | 143 (32.3%) | 300 (67.7%) | 0.2 | 160 (35.8%) | 287 (64.2%) | 0.2 |
| | Not at all | 24 (24.7%) | 73 (75.3%) | | 28 (28.9%) | 69 (71.1%) | |

| | | | | | | | |
|---|---|---|---|---|---|---|---|
| Number of meals containing animal protein | 100% of meals | 28 (40.6%) | 41 (59.4%) | 0.2 | 31 (44.9%) | 38 (55.1%) | 0.05 |
| | In 75% of meals | 85 (29.5%) | 203 (70.5%) | | 104 (35.7%) | 187 (64.3%) | |
| | Less often | 54 (29.5%) | 129 (70.5%) | | 53 (28.8%) | 131 (71.2%) | |
| Consumption of fruit and vegetables | Daily, at least in three meals | 19 (20.7%) | 73 (79.3%) | 0.07 | 22 (23.9%) | 70 (76.1%) | 0.05 |
| | Daily, at least in two meals | 88 (33.5%) | 175 (66.5%) | | 101 (38.0%) | 165 (62.0%) | |
| | Less often | 58 (31.7%) | 125 (68.3%) | | 64 (34.8%) | 120 (65.2%) | |
| Consumption of fruit and vegetables (2 categories) | Daily | 107 (30.1%) | 248 (69.9%) | 0.8 | 123 (34.4%) | 235 (65.6%) | 0.99 |
| | Less often | 58 (31.7%) | 125 (68.3%) | | 64 (34.8%) | 120 (65.2%) | |
| BMI (3 categories) | <20 | 32 (21.3%) | 118 (78.7%) | 0.001 | 34 (22.7%) | 116 (77.3%) | <0.001 |
| | 20–25 | 96 (31.7%) | 207 (68.3%) | | 113 (36.9%) | 193 (63.1%) | |
| | >25 | 37 (44.0%) | 47 (56.0%) | | 40 (47.1%) | 45 (52.9%) | |

**Part B: Quantitative Variables**
**Median and interquartile range (IQR)**

| Variable | AUDIT | | | AUDIT-C | | |
|---|---|---|---|---|---|---|
| | HAZ *n* = 167 | Low risk *n* = 373 | *p* | HAZ *n* = 188 | Low risk *n* = 356 | *p* |
| Age (years) | 19 (19–20) | 19 (19–20) | 0.1 | 19 (19–20) | 19 (19–20) | 0.3 |
| Weight (kg) | 70 (60–80) | 62 (55.3–71) | <0.001 | 70 (61.2–80) | 61 (55–70) | <0.001 |
| Height (cm) | 176 (168–182) | 170 (164–176) | <0.001 | 176 (169–182) | 169.7 (164–176) | <0.001 |
| BMI (kg/m$^2$) | 22.9 (2.4–24.7) | 21.5 (19.6–23.6) | <0.001 | 23 (2.5–24.7) | 21.4 (19.6–23.4) | <0.001 |
| Diastolic blood pressure (mmHg) | 73 (66–80) | 75 (67–80) | 0.2 | 74 (67.8–80) | 75 (67–80) | 0.7 |
| Dystolic blood pressure (mmHg) | 120 (111–127) | 119 (110–124) | 0.01 | 120 (111.8–128) | 118 (110–124) | <0.001 |
| AUDIT score | 10 (9–13) | 3 (2–5) | <0.001 | 10 (7–13) | 3 (2–5) | <0.001 |
| Hazardous Alcohol Use domain | 6 (5–7) | 3 (2–4) | <0.001 | 6 (5–7) | 3 (1–4) | <0.001 |
| Dependence Symptoms domain | 1 (1–2) | 0 (0–0) | <0.001 | 1 (0–2) | 0 (0–1) | <0.001 |
| Harmful Alcohol Use domain | 3 (2–5) | 0 (0–1) | <0.001 | 2 (1–4) | 0 (0–1) | <0.001 |
| BREF score | 79 (71–85) | 81 (73–87) | 0.06 | 80 (73–85) | 80 (72.8–87) | 0.7 |
| BREF physical domain | 18 (16.5–21) | 19 (17–22) | 0.08 | 19 (17–21) | 19 (17–21) | 0.9 |
| BREF psychological domain | 20 (18–23) | 21 (19–23) | 0.03 | 21 (18–23) | 21 (18–23) | 0.7 |
| BREF social domain | 12 (10–13) | 12 (10–13) | 0.3 | 12 (10–13) | 12 (10–13) | 0.5 |
| BREF environmental domain | 29 (26–31) | 29 (26–31) | 0.4 | 29 (26–31) | 29 (26–31) | 0.9 |

**Table S2.** Full version of the review of selected studies assessing alcohol use among students and doctors.

| Country and studied group | Year | G | *n* | Prevalence (%) | | | | AUDIT cut | | AUDIT score | | AUDIT-C | | |
|---|---|---|---|---|---|---|---|---|---|---|---|---|---|---|
| | | | | HAZ | SEX | HAR | smoke | HAZ | D | Me | Mn | HAZ | Me | Mn |
| Brazil [27] | - | S | 398 | 22.4 | - | - | - | 8 | 16 | - | | - | - | - |
| Finland [12] | 2007 | S | 465 | 33 | M: 49.0<br>F: 24.0 | - | 22.7 | - | - | - | - | M: 6<br>F: 5 | - | M: 5.5<br>F: 3.3 |
| France [21] | 2018 | P | 515 | 12.6 | F | 1.2 | 25.0 | 8 | 16 | 3 | - | M: 5<br>F: 4 | 3 | - |
| France [28] | 2011 | S | 171 | 11 | - | 21 | 21 [#]/16 ^ | 8 | 13 | - | - | - | - | - |
| France [9] | 2012–2013 | S | 198 | 31.9 | F | - | 16.0 | M: 7<br>F: 6 | 13 | T: 5<br>M: 5<br>F: 4 | T: 5.6<br>M: 6.2<br>F: 5.2 | - | - | - |
| German [29] | 2005 | S | 80 | 24 | M: 38.0<br>F: 15.0 | - | - | 8 | - | - | | - | - | - |
| Italy [3] | - | S<br>(S, N, P) | 641<br>(1500) | 6.1 | M: 4.2<br>F: 7.6<br>(F)<br>(M: 5.5<br>F: 7.1) | - | 16.6<br>(21.7) | - | - | - | - | M: 4<br>F: 3 | - | M: 1.5<br>F: 1.0<br>(M: 1.6<br>F: 1.1) |
| Korea [5] | 2016 | S | 323 | 45.5 | M | M: 18.4<br>F: 7.6<br>T: 13.6 | 18.3 | 8 | 16 | - | M: 9.8<br>F: 6.3<br>T: 8.1 | - | - | - |
| Korea [18] | 2016 | N | 922 | 44.6 [B] | M: 56.9<br>F: 34.1 | M: 11.2<br>F: 8.1<br>T: 9.5 [H] | 8.1 | M: 9<br>F: 6 | - | - | M: 12.8<br>F: 1.7 | - | - | - |
| Nepal [13] | 2017 | S | 588 | 47.8 * | - | 15.3 | 19.6 | - | - | - | - | - | - | - |
| Netherlands [2] | 2015–2016 | N | 5401 | 2.0 | M | - | - | 11 | - | - | - | M: 8<br>F: 7 | - | M: 6.2<br>F: 4.6<br>T: 5.2 |
| Paraguay [14] | - | S | 157 | 49 ** | F | - | - | - | - | - | - | M: 4<br>F: 3 | - | - |
| Poland [6] | 2011–2012 | S | 194 | 54.1 | M: 67.1<br>F: 5.0 | - | 19.6 | - | - | - | | M: 5<br>F: 4 | - | - |
| Poland [10] | - | S, N, P | 268 | 16.8 | - | 1.9 | - | 8 | 16 | - | - | - | - | - |
| Poland [4] | 2019 | S<br>(S, O) | 405<br>(877) | - | - | - | - | 8 | 22 | - | 17 | - | - | - |
| Poland [7] | 2010–2013 | S | 635 | 47.1 | M: 58.0<br>F: 4.5 | - | 17.8 | - | - | - | - | M:5<br>F:4 | - | - |
| Poland [11] | - | N | 500 | 65.0 | - | - | - | - | - | - | 11.2 | - | - | - |

| | | | | | | | | | | | | | | |
|---|---|---|---|---|---|---|---|---|---|---|---|---|---|---|
| Spain [39] | 2008–2011 | S | 192 | 49.5 | M: 5.9<br>F: 48.1 | - | 1.8 | M: 9<br>F: 6 | - | - | 6.5 | - | - | - |
| Sweden [15] | 2006 | S<br>(N) | 408<br>(342) | - | M | 17.2<br>(31) | - | 11 [&] | - | - | - | - | - | - |
| United Kingdom [17] | - | S | 244 | 47.1 | M: 54.3<br>F: 42.3 | - | - | 8 | - | - | - | - | - | - |
| United Kingdom [16] | 2014 | S<br>(S, N) | 820<br>(1136) | 4.5<br>(4.7) | - | 5.2<br>(6.9) | 3.5<br>(6) | 8 | 20 | - | - | - | - | - |
| United State of America [8] | 2004–2005 | S | 2710 | 18.1 | M | - | - | 8 | - | - | - | - | - | - |

G, group (target population); *n*, number of participants; HAZ, hazardous alcohol users; SEX, gender predominance of alcohol risky use; HAR, harmful alcohol users; D, dependence; Me, median; Mn, mean; S, medical students; N, other students; P, physicians O, others; M, male; F, female; T, total group; -, not available/not applicable; * alcohol users; [B] binge drinking; ** alcohol misuse/dependence; [H] heavy drinking; [#] daily smoker; ^ occasional smoker; [&] cutoff for harmful alcohol use.
